# Cytokine Receptor Endocytosis: New Kinase Activity-Dependent and -Independent Roles of PI3K

**DOI:** 10.3389/fendo.2017.00078

**Published:** 2017-05-01

**Authors:** Ping-hung Chen, Huiyu Yao, Lily Jun-shen Huang

**Affiliations:** ^1^Department of Cell Biology, University of Texas Southwestern Medical Center, Dallas, TX, USA

**Keywords:** cytokine receptor, endocytosis, endocytic pathway, receptor downregulation, PI3K

## Abstract

Type I and II cytokine receptors are cell surface sensors that bind cytokines in the extracellular environment and initiate intracellular signaling to control processes such as hematopoiesis, immune function, and cellular growth and development. One key mechanism that regulates signaling from cytokine receptors is through receptor endocytosis. In this mini-review, we describe recent advances in endocytic regulations of cytokine receptors, focusing on new paradigms by which PI3K controls receptor endocytosis through both kinase activity-dependent and -independent mechanisms. These advances underscore the notion that the p85 regulatory subunit of PI3K has functions beyond regulating PI3K kinase activity, and that PI3K plays both positive and negative roles in receptor signaling. On the one hand, the PI3K/Akt pathway controls various aspects downstream of cytokine receptors. On the other hand, it stimulates receptor endocytosis and downregulation, thus contributing to signaling attenuation.

## Introduction

Cytokines and cytokine receptors control a diverse spectrum of cellular functions that transcend specific organs and systems. A common theme of signaling from Type I and II cytokine receptors is that these receptors lack intrinsic enzymatic activities, and instead utilize cytokine-induced receptor oligomerization and conformational changes to drive activation of Janus tyrosine kinases (JAKs), which are constitutively bound to the intracellular domains of these receptors. Activated JAKs in turn phosphorylate tyrosine residues in the cytokine receptor intracellular domains, thereby creating a platform to recruit signaling proteins and elicit downstream signaling. The three major pathways activated by cytokine receptors are the JAK/STAT, the Ras/MAPK, and the PI3K/Akt pathways. These and other downstream pathways together control cell growth, differentiation, maturation, and apoptosis. Dysregulation of cytokine signaling results in human diseases and pathology (1–3).

Receptor endocytosis is a key regulatory mechanism that controls cytokine receptor signaling. This mini-review will focus on recent advances demonstrating new paradigms by which PI3K regulates cytokine receptor endocytosis. More general reviews of receptor endocytosis and PI3K signaling have been extensively covered in prior literature (4–13).

## Endocytosis and Signaling of Cytokine Receptors

Receptors are internalized from the plasma membrane into endocytic compartments, collectively called “endosomes” by endocytosis (8), which has long been recognized as a major mechanism to attenuate receptor signaling (14, 15). Constitutive (non-cytokine-induced) endocytosis regulates the number of receptors available on the cell surface to bind cytokines. Cytokine-induced endocytosis transports cytokine receptors to endosomes and subsequently to lysosomes for degradation, which terminates signaling by a process called “downregulation.”

Endocytosis also positively modifies receptor signaling. Endocytosis concentrates receptors in clathrin-coated pits or in endosomes, and the resulting increase in receptor density can promote receptor dimerization and activation (8, 16, 17). Examples of such activation include the enhanced activation of JAK/STAT signaling from granulocyte macrophage colony-stimulating factor (GM-CSF) receptors in clathrin-coated pits (18), and the requirement of endocytosis and concentration in endosomes for proper activation of JAK/STAT signaling by IL4 receptors (19). Endocytic compartments can also serve as signaling platforms to facilitate interaction of cytokine receptors with different signaling modules, thereby changing the signal output from those that occur at the plasma membrane (8, 16, 17). For example, although endocytosis of TNF receptors terminates NF-κB activation that occurs at the plasma membrane (20), it is also essential to promote the assembly of the death-inducing signaling complex in endosomes to drive apoptosis (21). TGFβ and BMP receptors interact in endosomes with adaptor proteins SMAD anchor for receptor activation and endofin to recruit downstream SMAD transcription factors (22).

Receptor signaling can also reciprocally regulate the endocytic machinery (23, 24). Endocytosis for many receptors is stimulated by receptor activation (25). Activation of receptors can increase the rate of *de novo* clathrin-coated pit formation (26) and can modulate the number of endosomes as well as regulate endosomal maturation (27, 28). A recent hierarchical map of genetic interactions in membrane trafficking also revealed new links between signaling and endocytic pathways (29). Therefore, endocytosis and signaling are intimately and bidirectionally linked. This coordination endows cells with the ability to resolve receptor signaling in space and time (11, 30).

### Multiple Pathways for Endocytosis of Cytokine Receptors

Receptor endocytosis is initiated at the plasma membrane and can be generally divided into clathrin-mediated endocytosis (CME) or clathrin-independent endocytosis (CIE) based on the involvement of the endocytic coat protein clathrin (31, 32). In CME, activated receptors recruit clathrin adaptors such as the AP2 complex, inducing the formation of a clathrin coat that stabilizes membrane curvature and drives invagination. Subsequently, vesicles are pinched off from the plasma membrane by the dynamin GTPase (10, 33). CIE is a composite of several distinct pathways, the best studied being the caveolin-mediated endocytosis (34, 35). These pathways, which can be either dynamin dependent or independent (13), require actin polymerization and either Src-family kinases in the case of caveolin-mediated endocytosis (36) or small GTPases such as RhoA and Rac1 for other CIE pathways (37).

Both CME and CIE are involved in endocytosis of cytokine receptors (15, 19, 38–40). CME mediates endocytosis of gp130, the shared receptor for IL6 family cytokines, and receptors for prolactin, thrombopoietin, erythropoietin, interferon, IL5 (IL5Rα), IL7 (IL7Rα), and IL36 (18, 39, 41–48). CIE mediates endocytosis of the common γ chain receptor, and IL2Rβ, IL4Rα, and IL15Rα receptors (19, 49–53). The same receptor can sometimes utilize both CME and CIE pathways. One example is endocytosis of the common β chain receptor (βc), which is shared by IL3, IL5, and GM-CSF receptors. βc co-localizes with both transferrin receptor (a CME marker) and cholera toxin-B (a CIE marker), but interestingly, signaling complexes mainly partition to the transferrin-containing fraction (51). The signaling dichotomy may involve intersectin 2, which is specifically involved in CME to regulate JAK2 and Akt activation downstream of βc (18). Growth hormone receptor also uses both CME and CIE for its internalization (44, 50), and perturbation of CIE specifically affects ERK activation downstream of the receptor but not STAT5 (54). Thus, differential use of CME and CIE may allow cells to regulate downstream signaling of cytokine receptors.

Ubiquitination plays an important role in receptor endocytosis through both CME and CIE (55). Through sequential actions of ubiquitin-activating (E1), ubiquitin-conjugating (E2), and ubiquitin-ligating (E3) enzymes, a small protein ubiquitin is covalently attached to lysine residues on target receptors. Because ubiquitin itself contains lysines that can serve as acceptor sites, target proteins can be subjected to mono-ubiquitination, multi-ubiquitination (mono-ubiquitination on multiple lysines), or poly-ubiquitination. Mono-ubiquitination has been shown to mediate protein trafficking and signaling (56), whereas poly-ubiquitination can promote protein degradation (55). Endocytic adaptor proteins and the endosomal sorting complex required for transport (ESCRT) contain ubiquitin-binding domain or ubiquitin-interacting motif (UIM), thereby facilitating their interaction with ubiquitinated receptors. This allows endocytic adaptors to target ubiquitinated receptors to the endocytic machinery and allows the ESCRT complexes to direct budding of ubiquitinated receptors into intraluminal vesicles within endosomes, thereby halting receptor signaling (57).

Endocytosis of cytokine receptors is regulated by ubiquitination. For example, ubiquitination by the E3 ubiquitin ligase SCF (βTrCP) drives endocytosis of growth hormone receptor, prolactin receptor, and the Type I interferon receptor (IFNAR1) (58–62). Another E3 ligase, c-Cbl, has been implicated in the internalization and/or degradation of the βc, thrombopoietin receptor, and the erythropoietin receptor (EpoR) (42, 63, 64). Interestingly, different ubiquitination sites on the EpoR are able to regulate distinct steps in the endocytic process (64).

### PI3K Pathway in Cytokine Signaling

Class IA PI3K is commonly activated by cytokine receptors (7). PI3Ks are lipid kinases that phosphorylate the 3′-hydroxyl group of phosphatidylinositol and its phosphorylated derivatives. At the plasma membrane, class IA PI3Ks phosphorylate phosphatidylinositol 4,5-bisphosphate [PI(4,5)P_2_] to generate phosphatidylinositol 3,4,5-triphosphate [PI(3,4,5)P_3_], which recruits PI(3,4,5)P_3_-binding proteins to activate downstream signaling. One of these downstream proteins is the serine/threonine kinase Akt, and together, the PI3K/AKT pathway regulates a plethora of cellular processes (4, 65, 66). The other is Rac1, which plays a major role in remodeling the actin cytoskeleton (5, 66).

Class IA PI3Ks function as heterodimers with a p110 catalytic subunit (p110α, β, or δ) and a p85-like regulatory subunit (p85α, β or their splice variants p55α, p50α, or p55γ) (4). p85 stabilizes and maintains p110 in an inhibited state and directly interacts with phosphorylated cytoplasmic tyrosines in cytokine receptors upon ligand binding. Conformational changes in p85 induced by receptor binding relieve its inhibition of p110 (67). Recent evidence suggests that the association and activation of PI3K by cytokine receptors promotes receptor endocytosis in addition to the activation of downstream Akt signaling (68, 69). Moreover, the contribution of the p85 regulatory subunit in these mechanisms can be PI3K kinase activity independent (48). Below, we discuss two new paradigms by which class IA PI3Ks regulate cytokine receptor endocytosis.

### PI3K and Actin-Mediated Endocytosis of IL2 Receptor (IL2R)

IL2 receptor belongs to the Type I cytokine receptors and is important for T cell immune function (70, 71). IL2R is composed of IL2Rα, IL2Rβ, and the common γ chain. Internalized IL2Rα recycles back to the plasma membrane, whereas IL2Rβ and the common γ chain are sorted to the lysosome and degraded (72, 73). IL2Rβ was among the first cytokine receptors shown to be internalized *via* CIE (74). It is constitutively internalized, but internalization is augmented by IL2 binding (69, 75).

Endocytosis of IL2Rβ is clathrin- and caveolin independent and relies on RhoA, dynamin, Rac1, PAK kinases (p21-activated kinases), and actin polymerization (38, 49, 76, 77). New studies showed that two rounds of actin polymerization are enlisted for IL2Rβ internalization. The first round relies on WAVE (WASP-family verprolin homologous protein), through a WAVE-interacting sequence in the cytoplasmic tail of IL2Rβ (78). This round of actin polymerization occurs before receptor clustering and is thought to be responsible for receptor recruitment near the base of membrane protrusion to initiate pit formation. The second round occurs just before receptor internalization and involves Pak1 phosphorylation of cortactin, another activator of actin polymerization (79, 80), thereby increasing its association with N-WASP (neuronal Wiskott–Aldrich syndrome protein) (77). Interestingly, dynamin, which mediates vesicle scission in the later stage of IL2Rβ internalization, also controls the transition of WAVE complex and N-WASP recruitments (78).

**Figure 1:**
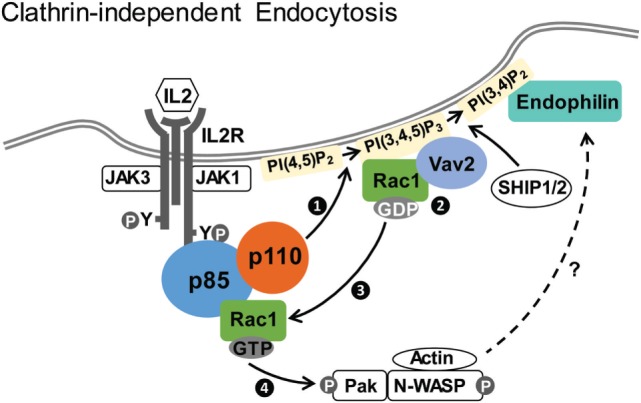
**Clathrin-independent endocytosis of IL2Rβ**. Upon IL2 stimulation, p85/p110 is recruited to IL2R and p110 is activated. Activated p110 generates PI(3,4,5)P_3_ (step 1), which recruits Vav2 and its substrate Rac1 (step 2). Vav2 facilitates conversion of GDP-bound Rac1 into the active GTP-bound Rac1 (step 3), which associates with p85 and stimulates the Pak1–cortactin–N-WASP cascade (step 4) to promote actin polymerization and endocytosis (69). Endophilin, recruited to PI(3,4)P_2_ generated from PI(3,4,5)P_3_ by SHIP1/2, is also required for IL2Rβ internalization (87).

Sauvonnet’s group showed that PI3K plays multiple roles in regulating IL2R CIE (69). First, IL2 stimulation activates PI3K, leading to the production of PI(3,4,5)P_3_ and the recruitment of Vav2, the guanine nucleotide exchange factor that activates Rac1 (81). Inhibitors of PI3K kinase activity, knockdown of p85 and Vav2, or overexpression of a mutant p85 devoid of p110-binding domain all inhibit IL2R endocytosis. Second, p85 binds directly to Rac1, with higher affinity for the GTP-bound active form. A model is thus proposed that IL2R activation of PI3K leads to the recruitment of both Vav2 and its substrate Rac1, which can stimulate the Rac1–Pak1–cortactin–N-WASP cascade to promote actin polymerization, driving IL2R internalization (Figure 1) (69). Because the WAVE complex is a known downstream effector of Rac GTPases (82, 83) and PIP_3_ (84, 85), PI3K may also regulate IL2Rβ CIE through WAVE.

Recently, endophilin and its interacting protein Alix (ALG-2-interacting protein X) have also been implicated in CIE of IL2Rβ (86, 87). Endophilin is a Bin/Amphiphysin/Rvs domain protein that is involved in vesicle endocytosis and membrane curvature generation (88, 89). This pathway, termed fast endophilin-mediated endocytosis (FEME) by the McMahon group, is utilized by IL2R as well as several G-protein-coupled receptors and bacterial Shiga and cholera toxins (87, 90). It is characterized by endophilin-positive uptake structures after ligand-induced receptor activation. Endophilin also works together with dynamin and actin in membrane scission (90, 91). As with the PI3K/Vav2 pathway described above, the FEME pathway depends on dynamin, Rac, Pak1, and actin polymerization (87), suggesting that FEME and PI3K/Vav2 mechanisms may be part of the same pathway. Importantly, PI3K kinase activity is required for FEME, because PI(3,4)P_2_, converted from PI(3,4,5)P_3_ by SHIP1/2-dependent dephosphorylation, is necessary for lamellipodin-dependent recruitment of endophilin in FEME (Figure 1) (87). The exact molecular details of this pathway, the degree to which the PI3K/Vav2 and FEME pathways are distinct or can be employed under different context, and whether PI3K regulates other aspects await future interrogations. In addition, whether other cytokine receptors can also utilize similar endocytic pathways is currently unclear.

## Cbl-Dependent Ubiquitination of p85 Mediates EpoR Endocytosis

**Figure 2:**
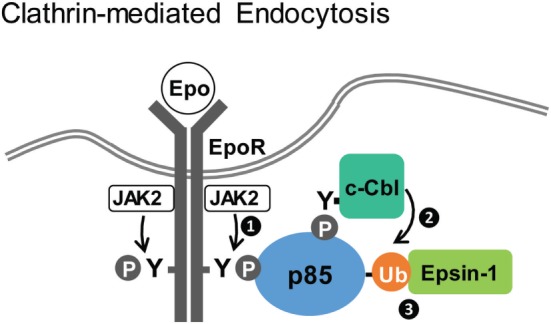
**Clathrin-dependent endocytosis of erythropoietin receptor (EpoR)**. Upon Epo stimulation, activated JAK2 phosphorylates EpoR cytoplasmic tyrosines to recruit p85 (step 1). Subsequently, ubiquitinated p85, mediated by c-Cbl (step 2), recruits Epsin-1 (step 3), linking EpoR to the endocytic machinery for downregulation (48, 68).

The EpoR is another member of the Type I cytokine receptors and is essential to drive red blood cell production (92, 93). In contrast to the IL2R, which forms heteromeric receptor complexes and associates with both JAK1 and JAK3 for signaling, EpoR forms homodimers and couples to only JAK2 for signaling. Epo-induced endocytosis is a key element in negative regulation of Epo signaling (48, 94) and controls cellular Epo sensitivity and the level of Epo in the circulation (95, 96). Studies in our laboratory have shown that Epo induces internalization of EpoR *via* CME, and we identified a novel function of p85 in EpoR endocytosis and downregulation (Figure 2) (48, 68). Epo stimulation activates JAK2, resulting in the phosphorylation of multiple EpoR cytoplasmic tyrosine residues, including Y^429^, Y^431^, and Y^479^. These phosphotyrosines serve as mutually redundantly docking sites for binding of the p85 subunit of PI3K to EpoR (48). p85 binding activates the catalytic p110 subunit, resulting in PI(3,4,5)P_3_ production and Akt signaling, which is required for erythroid differentiation. Unexpectedly, Epo-induced EpoR internalization does not require PI3K kinase activity (48). Instead, Epo-dependent ubiquitination of p85 by the E3 ligase c-Cbl recruits the endocytic adaptor protein, Epsin-1, through its UIM. Epsin-1 then connects the EpoR/p85 complex to the clathrin-mediated endocytic machinery for internalization (68).

The physiological relevance of this pathway is highlighted by mutated EpoRs found in patients with primary familial and congenital polycythemia (PFCP), a proliferative disorder of the red cell lineage characterized by increased red blood cell mass (97, 98). PFCP patients harbor mutations that delete the C-terminal cytosolic domain of the EpoR, resulting in EpoR truncations lacking all three tyrosines responsible for p85 binding. Mutated EpoRs mimicking those found in PFCP patients cannot bind p85 and are unable to recruit Epsin-1 to engage the endocytic machinery. As a result, these receptor variants do not internalize upon Epo stimulation and exhibit Epo hypersensitivity. Similarly, knockdown of Cbl also causes Epo hypersensitivity in primary erythroid progenitors. Restoring p85 binding to PFCP receptors rescues Epo-induced Epsin-1 co-localization and normalizes Epo hypersensitivity (48, 68). These results elucidate the molecular mechanism underlying Epo-induced p85-mediated EpoR internalization and demonstrate that defect in this pathway may contribute to the etiology of PFCP. Although still controversial, non-canonical heterodimeric complexes consisting of EpoR and the βc receptor have been implicated in non-hematopoietic tissues (99). Whether the p85–Cbl pathway plays a role in endocytosis of these complexes is unclear.

PI3K is activated by most cytokine receptors, whereas Cbl also functions downstream of many signaling receptors. Therefore, the p85–Cbl pathway might be utilized more broadly to contribute to endocytosis of other cytokine receptors. In addition, the same molecules may be employed in different ways for receptor endocytosis and downregulation. For example, the thrombopoietin receptor activates PI3K for signaling, and utilizes Cbl for downregulation. However, instead of ubiquitinating p85 as in the case of the EpoR, the thrombopoietin receptor itself is poly-ubiquitinated by Cbl upon stimulation, leading to its degradation (63).

## Conclusion and Perspectives

The two new paradigms reviewed here underscore the contribution of PI3K in CME (e.g., EpoR) as well as CIE (e.g., IL2R) of cytokine receptors. Besides class I PI3K discussed here, class II PI3K, which produces PI(3)P and PI(3,4)P_2_, has also been shown to participate in late stage CME (100). These broaden the roles of PI3K family kinases as fundamental and integral regulators of endocytosis in general.

The mechanisms underlying PI3K’s contributions are both kinase activity dependent and -independent. PI3K kinase activity is required to recruit Vav2 and endophilin for IL2R internalization. By contrast, in a PI3K kinase activity-independent manner, p85 recruits activated Rac1 to promote IL2R endocytosis and recruits Cbl/Epsin-1 to promote EpoR internalization. Therefore, PI3K plays both positive and negative roles upon cytokine receptor activation. On the one hand, the PI3K/Akt pathway controls various aspects downstream of cytokine receptors. On the other hand, it stimulates receptor endocytosis and downregulation, thus contributing to signaling attenuation.

These advances also highlight the emerging concept that p85 has functions beyond regulating PI3K kinase activity (101–105). For example, cytokinesis defects observed in p85α-deficient cells are restored by expression of a p85α mutant that does not bind p110 (102). It was also shown that p85 exhibits *in vitro* GTPase-activating protein (GAP) activity toward Rab5, which regulates vesicle trafficking and actin remodeling (106, 107). A p85α mutant with defective GAP activities perturbed PDGF receptor trafficking and caused cellular transformation *via* a kinase-independent mechanism (105, 108). Whether the GAP activity of p85 or Rab5 contributes to IL2Rβ or EpoR endocytosis is unclear. Moreover, p85 also interacts with dynamin (109), the contribution of this interaction is not known. Other p85-interacting proteins, such as phosphatases (e.g., SHP2) and adaptor proteins (e.g., IRS1), may also contribute to its function (110, 111).

One last layer of complexity we would like to bring up has risen from recent studies concerning dynamin isoform-specific functions. Normally, vertebrates express three dynamin (Dyn) isoforms: Dyn2 is ubiquitously expressed, whereas Dyn1 and Dyn3 are most highly expressed in specific tissues (112, 113). Under normal conditions, Dyn1 contributes little to CME in non-neuronal cells; however, Reis et al. recently showed that Akt, the canonical kinase downstream of PI3K, activates Dyn1 in epithelial cells to induce accelerated CME with altered dynamics (114). These results raise the interesting possibility that cytokine receptors may stimulate their endocytosis through Akt-dependent activation of Dyn1, adding to the concept that the endocytic machinery can be specifically adapted by signaling receptors to regulate their own endocytosis.

Regulatory controls of endocytic components and mechanisms significantly impact physiology and human diseases. Much of what we know about the cross talk between endocytosis and signaling comes from work done with model receptors such as receptor tyrosine kinases (RTK). Many of these lessons may translate to cytokine receptors, because JAK kinases activate many pathways in common with RTKs. Also, in many cases, JAK kinases are integral partner of cytokine receptors, making receptor/JAK complexes equivalent to RTKs (115, 116). However, signaling is not identical and differences are to be expected. Among the open questions are the following: First, do JAK kinases regulate endocytosis beyond receptor phosphorylation? Can they modulate the endocytic machinery directly? Second, does the PI3K/Akt signaling cascade provide a feedback loop for receptor endocytosis in general? Consistent with this notion, Akt promotes EGF receptor degradation by phosphorylating and activating the PIKfyve kinase (FYVE-containing phosphatidylinositol 3-phosphate 5-kinase), which stimulates vesicle trafficking to lysosomes (117). Third, does the GAP activity of p85 and/or other p85-interacting proteins play a role in cytokine receptor endocytosis? Fourth, how do cytokine receptors employ the molecular toolbox of signaling and endocytic proteins in different cell types and contexts such as normal vs. disease states? More detailed mechanisms are needed to understand the reciprocal cross talk between endocytosis and signaling, which will help to improve our understanding of the physiological functions of cytokine receptors.

## Author Contributions

All the authors contributed to the writing of the review.

## Conflict of Interest Statement

The authors declare that the research was conducted in the absence of any commercial or financial relationships that could be construed as a potential conflict of interest.
